# Supplementary material for: Pharmacokinetic Alteration of Paclitaxel by Ferulic Acid Derivative
Source: Pharmaceutics. 2019 Nov 9;11(11):593. doi: 10.3390/pharmaceutics11110593 (PMC6920777; doi:10.3390/pharmaceutics11110593)
Supplement: Supplementary file 1 [file pharmaceutics-11-00593-s001.pdf]

pharmaceutics

Supplementary materials: Pharmacokinetic Alteration of Paclitaxel by Ferulic Acid Derivative

Jaeok Lee, Song Wha Chae, LianJi Ma, So Yeon Lim, Sarah Alnajjar, Hea-Young Park Choo, Hwa Jeong Lee and Sandy Jeong Rhie

Table S1. The toxic effect of each derivative and VER in MCF-7/ADR cells after 2 h incubation.

Comp Conc. (μM)	5a	5b	5c	5d	5e	5f	5g	5h	VER
5	96.53 ± 0.55	100.65 ± 1.53	100.76 ± 3.32	101.74 ± 3.07	100.16 ± 0.57	98.92 ± 0.98	103.61 ± 1.97	103.46 ± 0.95	
10	94.09 ± 2.24	97.76 ± 0.74	98.50 ± 0.45	97.78 ± 1.44	99.36 ± 1.08	99.36 ± 1.73	101.70 ± 0.44	99.17 ± 0.32	
25	95.99 ± 2.78	96.10 ± 0.89	96.14 ± 0.58	97.07 ± 0.63	95.77 ± 3.53	100.16 ±1.47	100.44 ± 1.48	94.60 ± 1.81	
50	93.82 ± 1.67	96.10 ± 0.92	96.90 ± 1.81	97.14 ± 1.06	94.96 ± 3.05	96.89 ± 1.30	99.30 ±1.07	89.42 ± 2.36	
100	87.77 ± 3.50	92.05 ± 3.47	87.63 ±2.62	96.25 ± 2.44	94.84 ± 2.13	92.81 ± 0.86	101.72 ± 0.75	89.58 ± 3.04	99.80 ± 6.01

Data are presented as mean ± SD.

The numerical data were represented the cell survival ratio.
